# Based on molecular structures: Amyloid-β generation, clearance, toxicity and therapeutic strategies

**DOI:** 10.3389/fnmol.2022.927530

**Published:** 2022-08-31

**Authors:** Hai Yang, Jinping Li, Xiaoxiong Li, Linqiu Ma, Mingliang Hou, Huadong Zhou, Rui Zhou

**Affiliations:** ^1^Department of Neurology, Army Medical Center of PLA, Chongqing, China; ^2^Department of Neurology, The First Affiliated Hospital of Bengbu Medical College, Bengbu, China; ^3^Southwest Hospital, Army Medical University, Chongqing, China

**Keywords:** Alzheimer’s disease, amyloid-β, molecular structure, toxicity, therapeutic strategies

## Abstract

Amyloid-β (Aβ) has long been considered as one of the most important pathogenic factors in Alzheimer’s disease (AD), but the specific pathogenic mechanism of Aβ is still not completely understood. In recent years, the development of structural biology technology has led to new understandings about Aβ molecular structures, Aβ generation and clearance from the brain and peripheral tissues, and its pathological toxicity. The purpose of the review is to discuss Aβ metabolism and toxicity, and the therapeutic strategy of AD based on the latest progress in molecular structures of Aβ. The Aβ structure at the atomic level has been analyzed, which provides a new and refined perspective to comprehend the role of Aβ in AD and to formulate therapeutic strategies of AD.

## Background

Alzheimer’s disease (AD), which accounts for about 70% dementia, is the most common form of dementia. By 2050, global AD patients will triple in the number of 2010, with an incidence rate of 106.2/1 millions (Polanco et al., 2018). The pathological features of AD are amyloid plaques formed by amyloid-β (Aβ) deposition and neurofibrillary tangles formed by tau deposition (Hardy and Higgins, 1992; Sevigny et al., 2017). Current challenges in AD management include the lack of biomarkers that can be used for early diagnosis, and effective prevention and therapeutic strategies. The pathogenesis of AD involves abnormal metabolism and deposition of Aβ, hyperphosphorylation of tau, oxidative stress response, inflammatory changes of glia and microglia cells and other pathological events. Among them, the hypothesis of Aβ cascade is considered to be the most acceptable theory in the pathogenic mechanism of AD (Hardy and Higgins, 1992), although a few scholars doubt it (Wang, 2014; Herrup, 2015). In the past, Aβ was thought to be only associated with central nervous system (CNS) diseases such as AD or cerebral amyloid angiopathy (CAA), and scholars focused on its metabolic and toxic effects in the brain. However, more and more experimental results, epidemiological and clinical evidences show that the pathological role of Aβ is far beyond the CNS, and Aβ also has a toxicity in plasma, bone, and other peripheral organs (Li et al., 2014; Michno et al., 2021; Tian et al., 2021). In recent years, with the development of molecular biology techniques such as solid-state nuclear magnetic resonance (sNMR) and cryo-electron microscopy (Cryo-EM), the structure of Aβ was elucidated at the molecular and even atomic level, deepening the understanding of Aβ toxic mechanism. A detailed analysis of Aβ molecular structures and its toxicity mechanisms could improve current diagnostic and therapeutic strategies for AD and make it possible to design anti-Aβ drugs precisely. In the future, Aβ research may focus on some forms or structures of Aβ in the plasma or cerebrospinal fluid as a reliable indicator of the early diagnosis of AD; designing drugs or treatment strategies based on the molecular mechanism of Aβ oligomerization or fiber formation process and toxic effects; exploring the role of Aβ in the pathology of AD and treating AD beyond the CNS; exploring the toxic effects of Aβ on peripheral organs, such as whether it affects osteogenesis and osteoclasts of bone, whether it damages endothelial cells and smooth muscle cells of vessel, and whether its peripheral toxicity mechanism is also through the destruction of mitochondrial function and structure? Our review will describe the production, structure, and toxic effects in the brain, peripheral tissues, and plasma based on the different molecular structures and forms of Aβ, the relationship between Aβ and non-AD diseases, and possible therapeutic strategies for Aβ.

## Molecular structure of amyloid-β

Amyloid-β is a peptide consisting of 38–43 amino acid residues, mainly Aβ_42_ and Aβ_40_, which can induce toxicity by aggregating in the brain and other organs. Understanding the molecular structure of Aβ is of great significance for understanding the metabolic processes and pathological effects of various aggregation states of Aβ. All Aβ exists in three forms, including monomers, oligomers, and fibrils. Study indicated that Aβ_43_, an overlooked species, is potently amyloidogenic, neurotoxic and abundant *in vivo*. Also Aβ_43_ may play a major role in AD, as plaques contain more Aβ_43_ than Aβ_40_ (Welander et al., 2009).

### Monomer and oligomer structure

The natural Aβ_42_ and Aβ_40_ monomers are random coil structures or α-helix. During the formation of Aβ fibers, the monomer structure of Aβ_42_ and Aβ_40_ changes from natural structure to β-sheet (Rochet and Lansbury, 2000; Serpell et al., 2000; Sarroukh et al., 2011).

**FIGURE 1:**
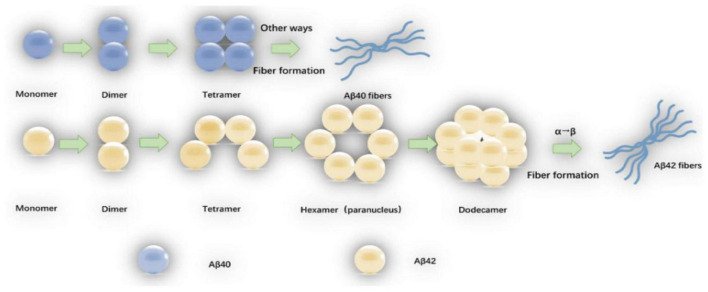
The oligomerization and fiber formation of Aβ_42_ and Aβ_40_: The monomers of Aβ_42_ and Aβ_40_ first form a dimmer, and a dimmer further forms a tetramer. The tetrameric Aβ_42_ structure (bending structure) is can be added with Aβ_42_ monomer or dimer to form Aβ_42_ hexamer (sub-nucleus). The two Aβ_42_ hexamers form a dodecamer (one stack paranuclear dimer). The natural structure of the Aβ_42_ monomer is converted to a β-sheet structure (the rate-limiting step in which the oligomer forms fibers) occurs at the 12-mer level. The tetrameric Aβ_*40*_ is in a ring structure, resisting further addition of Aβ_*40*_ monomer or dimer, fibers are also formed by other means.

Amyloid-β oligomers have a variety of structures which are formed spontaneously by folded or partially folded Aβ monomers. The Aβ oligomers under physiological conditions are at most 20-mer, and the tetramer is the determinant of the formation of hexamers and dodecamers (Nguyen and Derreumaux, 2014). Aβ_40_ exists only in the form of dimers, trimers, and tetramers, and the Aβ_40_ tetramer (ring structure) is tightly structured, resisting the addition of monomers or dimers, and does not form hexamers (sub-nuclei), but the Aβ40 fibers can be slowly formed by other means (Bitan et al., 2003; Bernstein et al., 2009). Aβ_42_ tetramer (bending structure) allows the addition of monomers or dimers to form pentamer and hexamer of a planar hexagonal structure, and two subnuclear hexamers are combined into a stacked sub-nuclear dimer [((Aβ42)6)2, dodecamer] (Chen and Glabe, 2006). The Aβ monomer structure change that α-helix converts to β-sheet (the rate limiting step in which the oligomer forms fibers) may occur on the Aβ_42_ dodecamer level (Bernstein et al., 2009). The oligomerization and fiber formation of Aβ42 and Aβ40 are shown in Figure 1.

**FIGURE 2:**
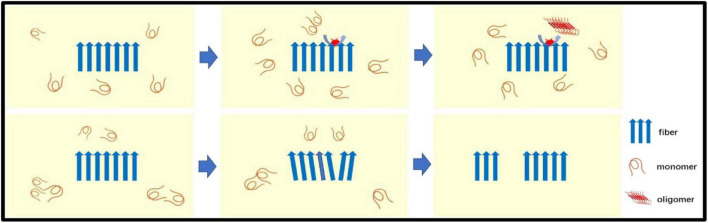
The secondary nucleation: Secondary nucleation includes two ways for catalyzing nucleation on the surface of Aβ fibers (top panel) and fiber breakage (bottom panel). Aβ monomer is catalyzed to form oligomers (new nuclei) on the surface of fibers. The Aβ fiber recruitment monomer is further extended by recruiting monomers and the ever-expanding fiber can be broken into two seeds (short segments) as a new aggregated nuclei.

Secondary nucleation involves two ways for catalyzing nucleation on the surface of Aβ fibers and fiber breakage (Figure 2). The aggregated Aβ monomer structure is partially unfolded, misfolded or inherently disordered. Some molecules must first be aggregated into the nucleus, and Aβ is then aggregated from the nucleus, so the formation of the nucleus determines the reaction rate. Experimental evidence indicates that such nuclei may be oligomers of different structural properties that differ from the final Aβ fiber structure. After the formation of the nucleus, the addition of the monomer becomes faster. There may be intermediates such as fibrils present. Catalyzing nucleation on the surface of Aβ fibers–the surface of Aβ fibers can catalyze the production of new nuclei. Fiber breakage-Aβ fiber recruitment monomer is further extended and the ever-expanding fibers can be broken into two short segments as new aggregated nuclei.

### Fiber structure

**FIGURE 3:**
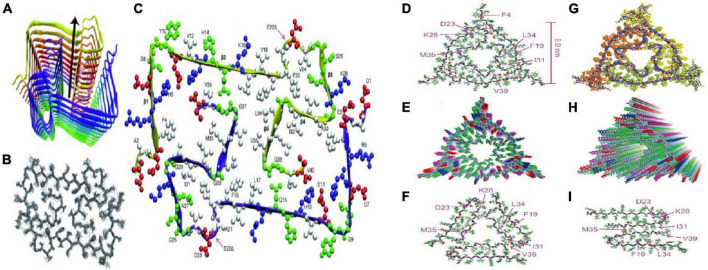
Two molecular structures of Aβ40 fibrils [figures coming from Lu et al. (2013) and Schutz et al. (2015)]. **(A)** Schematic view of the lowest-energy conformer of an Aβ1-40 E22Δ bi-decamer (2 × 10 monomers). The symmetry axis (arrow) coincides with the long axis of the fibril. **(B)** NMR bundle of the middle layer only. **(C)** Cross section of the fibril hydrophobic residues is colored white, negatively charged residues red, positively charged residues blue, and polar ones (and Gly) green. **(D)** Structure with the lowest total experimental restraint energy in Xplor-NIH calculations. The threefold-symmetric repeat unit is shown, as viewed along the fibril growth axis. Backbone and sidechain carbon atoms are gray and green, respectively. **(E)** Superposition of 20 structures that are consistent with experimental restraints (PDB code 2M4J). Sidechains of the three Aβ40 molecules in the repeat unit are yellow, green, or orange. **(F,G)** Two views of the idealized fibril structure, created by repeating the trimeric unit 18 times with 0.48 nm displacements along the fibril axis. **(H,I)** Structural models for Aβ40 fibril polymorphs with threefold and twofold symmetry about the fibril growth axis, developed previously from solid-state NMR and electron microscopy measurements on fibrils grown *in vitro*.

Petkova et al. (2002) analyzed Aβ_40_ fibers by sNMR and X-ray diffraction: (1) amino acid residues 12–24 and 30–40 adopt β-strand conformations and form parallel β-sheets by intermolecular hydrogen bonding; (2) the 25–29 residue contains a bended peptide backbone that brings two β-sheets to the interaction via the sidechain-sidechain; (3) the single cross-β unit is a double-layer β-sheet structure having a hydrophobic core and surface; (4) the only charged side chains in its hydrophobic core are D23 and K28, which form a salt bridges between them; (5) fibers with minimum mass length and diameter are composed of two cross-β units, with hydrophobic surfaces juxtaposed. In addition, Bertini et al. (2011) found that the addition of new inter-monomers occurred at the protofilament interface during the elongation of Aβ_40_ fiber. Lu et al. (2013) and Schutz et al. (2015) obtained Aβ_40_ fiber structures in their respective sNMR studies (Figure 3), these fiber structures have some the commonalities. Residue 1–10 structure is disordered, residues 12–24 and 30–40 constitute two β-chains and form a β-sheet by intermolecular hydrogen bonding, and residues 25–29 constitute a curved portion of the peptide backbone and the two β-strands are in contact through the interaction between the side chains.

**FIGURE 4:**
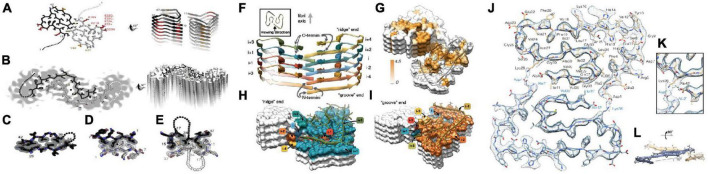
Two molecular models of Aβ42 fibrils architecture and atomic model of the fibril cross section [figures coming from Eisenberg and Sawaya (2016) and Gremer et al. (2017)]. **(A)** The near-atomic resolution model determined by solid-state NMR by Wälti et al. Two double-horseshoe–shaped molecules of amyloid-β(1–42) are shown (black and gray) related by a twofold axis (marked by a circle), which runs down the center of the fibril. The N-terminal 14 residues are disordered; one possible conformation is shown here by dotted lines. Many of the known familial mutations are carried by residues located on the outer surface (red). The surface hydrophobic patch formed by residues V40 and A42 (orange) may explain the greater rate of secondary nucleation by the 1–42 species compared with 1–40. **(B)**. The lower resolution (5–7 Å) model determined by Schmidt et al. by cryo-EM is another polymorph of amyloid-β(1–42), also with two molecules per layer, related by a twofold axis, and also with a poorly ordered N terminus. The gray color represents a slice through the cryo-EM map. The two molecules appear to be related by a homo-steric zipper type of bonding. In both A and B, the models are viewed down the fibril axis on the left and nearly perpendicular to the fibril axis on the right. **(C)** A hetero-zipper in amyloid-β(1–42) fibrils from Wälti et al. **(D)** A homo-zipper from GNNQQNY. **(E)** A noncontiguous homo-zipper from Wälti et al. Dotted lines represent intervening residues. **(F)** Side view of the atomic model showing the staggered arrangement of the non-planar subunits. **(G)** Surface representation of a fragment of the atomic fibril model. The surface is colored according to hydrophobicity (Kyte-Doolittle scale) [gradient from brown (hydrophobic, 4.5) to white (neutral, 0.0)]. View of the “ridge” **(H)** and “groove” **(I)** fibril ends. **(J)** Two subunits, one from each protofilament, are shown (blue and brown) together with the masked EM density map. **(K)** Detailed view of the interactions between the N- and C-terminus and the sidechain of Lys28 (at contour level of 1 σ). **(L)** Side view of the same two opposing subunits showing the relative orientation of the non-planar subunits.

Schmidt et al. (2015) analyzed the characteristics of Aβ_42_ fiber structure with Cryo-EM: (1) peptide dimers packed face to face form the individual layers of the Aβ_42_ fibril; (2) these two peptides form a dimer with the same tilde-shaped conformation and interaction through the hydrophobic C-terminal β-strands package; (3) the C-terminus of the peptide is close to the fibril axis, creating a hydrophobic core on the fibril axis, surrounded by a structurally more flexible, charged peptide N-terminal segment. Colvin et al. (2016) and Walti et al. (2016) resolved the spatial structure of residues 15–42 in Aβ_42_ fibers by Snmr, respectively. It is believed that the fiber polymorph is mainly affected by the C-terminus and the two protofilament in the fiber are 2^1^-spiral symmetry. Gremer et al. (2017) analyzed the molecular structure of all residues of Aβ_42_ fiber by Cryo-EM, and proposed that six Aβ_42_ monomers constitute the smallest fiber unit, in which each two monomers constitute a dimer plane, and the existence of the inter-ion ionic bond in the Aβ_42_ fiber and the “LS” conformation of the monomer in the Aβ_42_ fiber were clearly confirmed (the N-terminus is L-form and the C-terminus is S-type) (Figure 4).

## The relationship between molecular structure and generation of amyloid-β

Amyloid-β is a regulated cleavage product of amyloid precursor protein (APP), so APP metabolism has a direct effect on Aβ generation. APP mainly has three isomers, which are APP_695_, APP_751_, and APP_770_. APP695 is mainly expressed in neurons, while APP695 and APP751 is mainly expressed in glial cells and other peripheral cells, APP770 is expressed in vascular endothelial cells. Aβ_42_ and Aβ_40_ are main components of senior plaques within the brain of AD and are regarded to as an initiating factor for the pathogenesis of AD (Del Turco et al., 2016). Closely related to the onset of AD, Aβ_42_ is present in the brain as a physiological factor, as well as Aβ_40_. There are more Aβ_40_ in AD brain than Aβ_42_, but Aβ_42_ is more toxic than Aβ_40_ (Haass and Selkoe, 2007).

**FIGURE 5:**
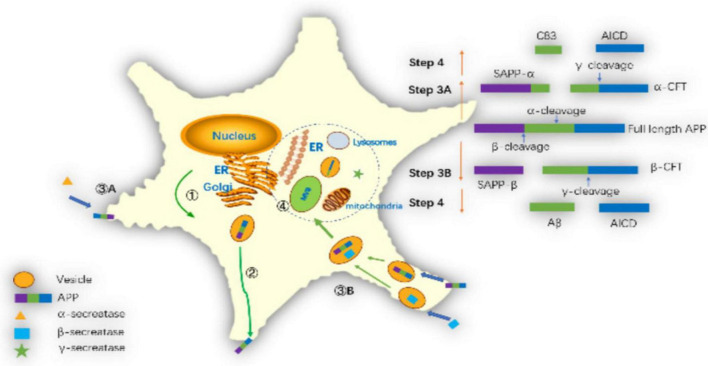
Pathways of amyloid precursor protein (APP) processing and amyloid-β (Aβ) generation: ➀ APP further modifies the endoplasmic reticulum and Golgi and synthesizes it via the endoplasmic reticulum. ➁ APP transports to the cell membrane through vesicles. ➂A The α-secretase cleaves APP on the cell membrane as sAPP_α_ and C_83_. ➂B The β-secretase cleaves APP into sAPP_β_ and C_99_ in the endosomes. ➃ The γ-secretase complex cleaves C_99_ into AICD and Aβ in mitochondrial membrane, multivesicular body or lysosome membrane.

Amyloid precursor protein is cleaved in different subcellular regions, eventually producing Aβ_42_ or Aβ_40_. Pathways of amyloid precursor protein (APP) processing and amyloid-β (Aβ) generation are shown in Figure 5. Zhang et al. (2011) summarized the basic steps. Firstly, after translation in the endoplasmic reticulum (ER) APP is further modified in the ER and Golgi. Secondly, APP is transported to the cell membrane by vesicles. Thirdly, APP is cut in two ways on the cell membrane. One way is non-amyloid protein pathway, APP is cleaved by α-secretase on the membrane to generate soluble APP_α_ and α secretase C-terminal fragment (C_83_). Another way is amyloid pathway, β-secretase cleaves APP into soluble APP_β_ (sAPP_β_) and β secretase C-terminal fragment (C_99_) in endosomes. Fourthly, γ-secretase complex cleaves C_99_ into the APP intracellular domain (AICD) and Aβ. In recent years, there has been a new understanding of the process of Aβ generation. Firstly, it was previously thought that β-secretase cleaves APP on the cell membrane, but Das et al. (2013) found that APP and β-secretase were internalized into the cell to form endosomes, and then fuse in the endosomes, and then cut the APP into sAPP_β_ and C_99_ by β-secretase in 2013. Secondly, it was thought that the γ-secretase cleavage site was on the cell membrane in the past, but Area-Gomez et al. (2009, 2012) found that the γ-secretase cleavage might occur in mitochondrial membrane, multivesicular body and lysosome in 2012. Thirdly, it was previously thought that C_83_ could not generate Aβ, but Siegel et al. (2017) found that C_83_ generated a small amount of Aβ in 2017.

## The relationship between molecular structure and toxicity of amyloid-β

### Toxicity of amyloid-β fiber

Amyloid-β fiber mainly induces toxicity through four ways. Firstly, three hydrophobic groups consisting of Val18-AlA21, Lys28, and Val40-ALA42 (only Aβ42) are tightly bound by hydrophobic interactions, extending along the surface of Aβ fibers, which may affect cell viability; secondly, formed Aβ fiber breakage produces two short fragments for replication, resulting in more Aβ fiber formation; Thirdly, Aβ peptide is imported into mitochondria via the TOM import machinery and localized to mitochondrial cristae (Hansson Petersen et al., 2008). Aβ fibers bind to the mitochondrial membrane, interfering with the function of the oxidative respiratory chain type I complex. In addition, a considerable amount of Aβ is produced at mitochondria ER contact sites including outer mitochondrial membrane and mitochondria-associated ER membranes. Enhanced Aβ production at this site may disturb ER, mitochondrial and mitochondria-ER contact site function (Schreiner et al., 2015). Fourthly, Aβ fiber surface catalyzes the formation of new Aβ oligomers, generating greater toxic effects (Rhein et al., 2009; Cohen et al., 2013; Riek and Eisenberg, 2016).

### Toxicity of amyloid-β oligomers

**FIGURE 6:**
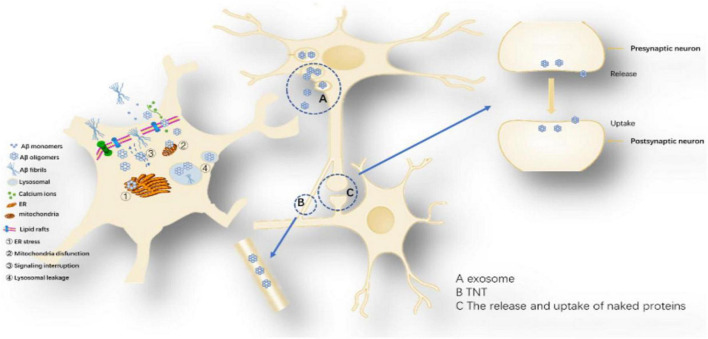
Proposed toxicity mechanisms caused by Aβ oligomers: The toxicity mechanism mainly includes two aspects, one is the cell-to-cell transmission, and the other is the influence on the intracellular system. Cell-to-cell transmission includes (A) Secretory vesicle mechanism, (B) Intermembrane bridge Mechanisms and (C) The release and uptake of naked proteins. The influence on the intracellular system includes ➀ ER stress, ➁ Mitochondria disfunction, ➂ Signaling interruption, and ➃ Lysosomal leakage.

Amyloid-β oligomers are short polymers in which a small amount of Aβ monomers are linked by covalent bond repeats, and their toxic effects have been well confirmed (Figure 6) (Lambert et al., 1998; Hsia et al., 1999). Endogenous Aβ oligomers are 1,000 times more toxic than synthetic soluble Aβ monomers (Bateman et al., 2019). Aβ dimer accumulates in lipid rafts (lipid regions of cell membranes), promotes Aβ aggregation and plaque formation, and its accumulation time is consistent with the time of memory loss, impairing memory function (Kawarabayashi et al., 2004; Shankar et al., 2008; Hong et al., 2014). Aβ trimers have the strongest ability to attenuate long-term potentiation (LTP) and can trigger abnormal cell signaling to reduce electrical activity in hippocampal synapses (Townsend et al., 2006; Talantova et al., 2013). We know that the dimers, trimers, and tetramers of Aβ are shared by Aβ_40_ and Aβ_42_, and the toxicity of pentameric and above structures is only Aβ_42_. The hollow tube structure (C-terminal outward) of Aβ_42_ hexa- or 12-mer can form a cyclic structure on the membrane to destroy its integrity, which can damage the memory function of AD mice, leading to neuronal death and neurodegeneration (Lesne et al., 2006; Bernstein et al., 2009; Miller et al., 2010).

Amyloid-β oligomerization internalization triggers intracellular systemic damage, increases ER stress levels, mediates calcium imbalance leading to decreased cell viability and activation of apoptotic caspase 3 (Resende et al., 2008; Umeda et al., 2011; Economou et al., 2016). Aβ oligomers can disrupt endosome vesicles and interfere with the retrograde transport of brain-derived neurotrophic factors, downregulating ubiquitin C-terminal hydrolase L1 (UCH-L1) and deubiquitinating enzymes, interfering with proteolysis (Poon et al., 2013). Aβ_42_ and Aβ_40_ oligomers can undergo intercellular transfer through the endosomal transport system and are highly metastatic with Aβ_42_ (Domert et al., 2014). Since Aβ oligomers are in the dynamic equilibrium of continuous formation and degradation, there is no authoritative literature report on the content and presence of various oligomers (Noguchi et al., 2009; Esparza et al., 2016). However, current studies suggest that Aβ_42_ hexamers and dodecamers are the most neurotoxic isoforms.

Accumulation of beta-amyloid protein (Aβ) in the extracellular space of the brain has been hypothesized to be a culprit in the pathogenesis of AD. In this issue of Neuron, Cirrito et al. (2008) describe a series of experiments demonstrating that extracellular Aβ levels are directly modulated by neuronal and synaptic activity (Schroeder and Koo, 2005).

Possible mechanisms of Aβ cell delivery: (1) Secretory vesicle mechanism: promotes the transfer of Aβ from a neuron across the synapse or more distantly to other neurons, exerting its toxic effects (Candelario and Steindler, 2014). Several observations support the idea that some of the Aβ is released via exosomes (Rajendran et al., 2006). One study showed that tunneling nanotubes (TNTs) formed by various neuronal cell lines can mediate the transport of different forms of Aβ and that this transport is bidirectional, with different velocities in various cell lines (Rustom et al., 2004; Zhang et al., 2021). Amyloid-β induced membrane damage by TNT (Dilna et al., 2021); (2) Release and uptake of Aβ: Aβ oligomers may be similar to Tau protein and α-synuclein, leaking in the pre-synaptic or posterior space and being directly ingested by another neuron (Hampel et al., 2021). Uptake, degradation and release of Aβ are also possible in inflammation-responsive cells of the CNS (Chung et al., 1999; Maler et al., 2009).

### The damage of amyloid-β oligomers and fibers to mitochondria

Neurons are highly dependent on mitochondria to produce energy, so mitochondrial damage has a great impact on neuronal function (Fang et al., 2019). Aβ oligomers and fibers can impair mitochondrial function by the way of oxidative phosphorylation, mitochondrial dynamics (transport, fission, and fusion), bioenergetics and mitochondrial phagocytosis (removal of damaged mitochondria by autophagy). Aβ binds metal ions (mainly Cu^2+^, Fe^3+^) to reduce the formation of reductants (mainly Cu^+^, Fe^2+^), resulting in lipid and protein peroxidation, which causes oxidative stress (Smith et al., 2007). Aβ, similar to other pore forming toxins, induces perforation of neuronal membranes and mitochondrial membrane causing an increase in membrane conductance, intracellular calcium influx. Aβ oligomers pores on the mitochondrial membrane, causing Ca^2+^ non-regulating influx, causing multiple Ca^2+^ signaling pathways to be disordered, leading to neuronal death (Lau et al., 2007). One study demonstrated that blocking the MCU complex (is the main pathway for mitochondrial Ca^2+^ influx) *in vivo* with Ru360–a specific blocker of the channel–prevented the mitochondrial Ca^2+^ uptake elicited by TgCM. Then they observed that Ru360 did not interfere with the rise in the cytosolic Ca^2+^ induced by Aβ. These results suggest that MCU is required for the increase in mitochondrial Ca^2+^ induced by Aβ *in vivo*, and points to MCU as a potential target candidate for AD (Calvo-Rodriguez and Bacskai, 2020).

### Inflammatory damage caused by amyloid-β oligomers and fibers

A variety of inflammatory factors secreted by microglia activated by Aβ oligomers or fibers not only cause inflammatory damage to neurons, but also increase APP expression and Aβ deposition (Blasko et al., 2000; Perry et al., 2010). Activated microglia release high mobility group protein-1 (HMG1) to bind to Aβ to form oligomers, while HMG1 is positively fed back to microglia to reduce its Aβ scavenging capacity (Takata et al., 2003; Hickman et al., 2008). Apoptosis-associated speck-like protein containing a C-terminal caspase activation and recruitment domain (ASC) spots released by microglia can rapidly bind to Aβ, increasing the formation of Aβ oligomers and Aβ aggregation (Venegas et al., 2017; Hulse and Bhaskar, 2022). Some studies found the nod-like receptor family pyrin domain containing 3 (NLRP3) inflammasome is activated by glycolytic enzymes (Hughes and O’Neill, 2018); activation of the NLRP3 inflammasome has been demonstrated to contribute to AD pathology (Ising et al., 2019). However, there is no consensus on the difference in the effects of Aβ_42_ or Aβ_40_ on microglia. In addition, activated microglia can mediate tau hyperphosphorylation via Toll-like receptor 4 and IL-1 receptors (Bhaskar et al., 2010).

### Loss of amyloid-β physiological effects

Overexpression of Aβ and APP are very important pathological proteins in AD, but Aβ and APP have some physiological functions under normal circumstances (Pearson and Peers, 2006; Puzzo et al., 2008). APP is a transmembrane glycoprotein that plays an important role in neurogenesis, cellular stress, synaptic function, and plasticity. Soluble APPα mediates voltage-gated calcium channels to maintain calcium homeostasis under stress conditions. Aβ generated under physiological conditions can enhance synaptic activation efficiency and plasticity (Kim et al., 2013; Li et al., 2013), and its generation is directly proportional to neuronal activity (Cirrito et al., 2008). Aβ can bind and transport cholesterol and participate in lipid regulation (Umeda et al., 2010). Soluble Aβ has a physiological role in regulating synaptic function and promoting neuronal growth and survival (Puzzo et al., 2015). There is evidence that Aβ protects the brain from infection, repairs blood–brain barrier (BBB) leakage, promotes damage recovery and regulates synaptic function, and the cellular generation of Aβ rapidly increases in response to a physiological challenge and often decreases on recovery (Yu et al., 2018). In the process of Aβ oligomerization, fibrosis and deposition into amyloid plaques, soluble Aβ loses its normal physiological function which may be one of the AD pathological mechanisms.

## Tau molecular structure and toxicity linked to amyloid-β

The neurofibrillary tangles formed by phosphorylated Tau are another important pathological marker of AD, but phosphorylated Tau is not all toxic, only the phosphorylation of specific residues AT180 (Tr231 and SR235) or 12E8 (Ser262 and Ser356) is pathologic, and phosphorylated Tau can reduce hippocampal excitability (Hatch et al., 2017). By analyzing the molecular structure of Tau, it was found that both tau and Aβ are amyloid proteins and can form amyloid fibrils. Kadavath et al. (2015) used sNMR to find that Tau fiber residues 269–284 and 300–312 are in a hairpin conformation, which is markedly different from Tau on the microtubule. Further study found that Tau fibers interact with hydrophobic and polar bonds to form antiparallel and paired β-folds, and the N-terminal of the β-fold is formed by hexa-peptides 306–311 (VQVYK), which is essential for Tau polymerization into fibers (Xie et al., 2015). Fitzpatrick et al. (2017) used Cryo-EM to find that the various isomers of Tau were aggregated through the core region and the monomers were randomly added, Tau fiber adopts β-fold structure and is closed by G355. Acetylation of Tau lysine residues (such as Lys174 and Lys280) inhibits tau degradation, impairs the interaction of tau with microtubules, promotes tau aggregation, and disrupts synaptic signaling (reduced KiBRA, a memory-related protein), leading to cognitive deficits (Min et al., 2010, 2015; Cohen et al., 2011; Irwin et al., 2012; Tracy et al., 2016). Studies on toxic effects have found that Tau, like Aβ, exerts toxic effects primarily through forms. Tau oligomers can lead to electrophysiological damage and structural degradation of neurons, damage the function of microtubules or molecular motors and thus affect axon transport, and also cause intercellular transmission and corresponding changes of Tau protein in receptor neurons (Kopeikina et al., 2011; Calafate et al., 2015). On the other hand, Tau has also been found to have secondary nucleation. It has been found that the hexamer Tau has a strong ability to aggregate fibers (Jackson et al., 2016). In addition, Tau can also work with Aβ to produce toxicity (Ittner et al., 2010). The hippocampus of tau knockout mice are resistant to ATP oligomer-induced LTP damage, indicating that Tau mediates the damage process of Aβ to LTP (Li and Gotz, 2017; Puzzo et al., 2017). Aβ oligomers form a complex with synaptic NMDA and mediate synaptic toxicity of tau via the CaMKK2-AMP kinase pathway (Mairet-Coello et al., 2013).

## Structure forms and generation, clearance of amyloid-β in brain

In the brain, Aβ is mainly generated by neurons and then secreted into the intercellular space. Aβ is mostly secreted on the axon (pre-synaptic) and a little on the dendrites (post-synaptic). Astrocytes and microglias also generate small amounts of Aβ (Wegiel et al., 2001; Niederst et al., 2015). The type of Aβ in the brain is mainly Aβ_40_, followed by Aβ_42_.

Clearance of Aβ in the brain is mainly extracellular and intracellular. Intracellular clearance is mainly degraded by enzymes in the intracellular proteasome and lysosome systems of neurons and glial cells (Guenette, 2003). Intracellular Aβ is degraded by the ubiquitin-proteasome pathway, lysosomal cathepsin and autophagy pathways (Zhang et al., 2009; Querfurth and LaFerla, 2010; Perez et al., 2014). The intracellular degradation of Aβ is affected by four major factors: enzyme expression and activity, ligand affinity and competition, cellular uptake capacity, and initiation of intracellular degradation pathways, all of which are found to be impaired in the context of AD (Wang et al., 2003; Lucin and Wyss-Coray, 2009). Deubiquitinase is one of the important Aβ degradation pathways in neuron, and the Aβ accumulation inhibits the deubiquitinase activity and affects Aβ degradation (Zhang et al., 2018). Lysosomes contain hydrolytic enzymes that are capable of degrading Aβ, whereas the hydrolytic machinery in AD are often overwhelmed. As the amount of Aβ exceeds the lysosomal degradation ability, it will aggregate in the lysosome or leak into the cytoplasm (Umeda et al., 2011; Gowrishankar et al., 2015). There are three types of autophagy, namely macroautophagy, microautophagy and chaperone-mediated autophagy, in which macroautophagy and chaperone-mediated autophagy dysfunction are associated with AD (Zare-Shahabadi et al., 2015). A large accumulation of Aβ-rich unprocessed autophagic vacuoles was found in the neurites of AD, indicating significant impairment of autophagy degradation (Nixon, 2007; Boland et al., 2008). One study has suggested that defects in the autophagy-lysosomal pathway may even occur before Aβ aggregation (Correia et al., 2015).

There are five main ways of extracellular clearance: First, Aβ degrading by related enzymes. Animal experiments and clinical studies have suggested that serum Aβ levels are elevated, enkephalinase (NEP) levels is decreased (Yasojima et al., 2001; Yin et al., 2006; Lim et al., 2011); Second, the phagocytic degradation of Aβ glial cells. Aβ in brain interstitial fluid (ISF) is phagocytized by microglia and astrocytes (Yoon and Jo, 2012); Aβ plaques are degraded by microglia and astrocytes and degraded by the autophagy-lysosomal pathway (Cho et al., 2014; Morrone et al., 2015). It was found in AD that Aβ phagocytized by glial cells was re-released extracellularly, indicating impaired phagocytic ability of glial cells (Nagele et al., 2003; Lee and Landreth, 2010). In AD, the increase in Aβ degraded by the endosomal-lysosomal pathway leads to the accumulation of autophagic vacuoles and abnormal expansion of endosomes, leading to dysfunction of whole-cell proteasomes (Nixon and Cataldo, 2006; Chesser et al., 2013; Norambuena et al., 2018); Third, Aβ transport in BBB. One study found that Aβ generation rate did not differ between AD and control group, while the Aβ clearance rate across the BBB decreased by 30%, indicating that the BBB changes play a role in AD (Mawuenyega et al., 2010). Low density lipoprotein receptor-related protein-1 (LRP1) is widely expressed in the BBB endothelial cells, and transports Aβ in the brain to the plasma to play a role in clearing Aβ in the brain (Erickson et al., 2012). Receptor for advanced glycation endproducts (RAGE) is a receptor on the BBB endothelial cells, which transports Aβ in the plasma to the brain, which is normally low in expression levels (Chen et al., 2014). LRP1 and RAGE together regulate Aβ transport in the BBB and maintain Aβ balance in the brain. BBB damage may attenuate the expression of LRP1 and cause clearance of Aβ, and may also increase the expression of RAGE, increase the Aβ transported into the brain, and promote the progression of AD (Jaya Prasanthi et al., 2008; Zlokovic, 2011); Fourth, the blood cerebrospinal fluid barrier transport of Aβ. In the case of AD, the expression of LRP1 and RAGE is completely opposite to that in the case of non-AD, which may be the compensation of the body for b BBB transport disorders (Johanson et al., 2008; Wraith and Nicholson, 2012; Gonzalez-Marrero et al., 2015); Fifth, astrocyte-mediated intracellular lymphatic system (ISF bulk flow). It was previously thought that this removal only accounted for about 10%, and it is now found that its proportion may be higher (Iliff et al., 2012; Kress et al., 2014).

## Generation, structure form and clearance of amyloid-β in blood circulation

### Generation and structure forms of amyloid-β present in blood circulation

Aβ_40_ and Aβ_42_ in plasma are present in monomeric form and do not form fibers. Albumin, erythrocyte membrane proteins and other plasma molecules bind more than 90% of Aβ monomers (Biere et al., 1996; Kuo et al., 1999, 2000a,b), while the half-life of unbound Aβ_40_ and Aβ_42_ is only 2.5–3 min in plasma (Ghiso et al., 2004). Aβ_40_ accounts for about 80% of the total plasma Aβ, and Aβ_42_ accounts for less than 10% (Chen et al., 1995; Zheng et al., 2012). In the case of AD, the Aβ monomer structure in plasma was found to change from a natural structure (random coil or α-helix structure) to a β-sheet structure and it has been reported that the risk of AD can be assessed by detecting changes of Aβ monomer structure in plasma (Nabers et al., 2016, 2018). APP_751_ and APP_695_ are abundantly expressed in platelets and 95% Aβ in the plasma is derived from platelets (Sonkar et al., 2014). Peripheral tissue can also produce Aβ into the plasma, affecting Aβ level in the plasma (Kuo et al., 2000a,b).

### Clearance and structure forms of amyloid-β in blood circulation

The Aβ removal is mainly liver-based in the plasma, and more than 90% Aβ is excreted into the bile by liver cells (Sehgal et al., 2012). The data obtained by the experiments showed that under normal conditions, the daily excretion of intact Aβ in urine was less than 1% of the circulating pool (Ghiso et al., 1997).

#### Amyloid-β monomer clearance in liver

Under normal conditions, the liver is the main organ of plasma Aβ_40_ clearance. In a treatment study, it was found that the ability of the liver to clear Aβ directly affects plasma Aβ and intracerebral Aβ levels. This study using Withania somnifera to treat AD found that Withania somnifera is an extract from plant roots that significantly increases the expression of LRP1 and NEP in the liver, the level of plasma LRP1 and the ability to bind Aβ, thereby increasing the clearance of Aβ in the brain. This suggests that in AD, the ability of the liver to clear Aβ is reduced, which may affect Aβ levels in the plasma and increase the risk of AD (Sehgal et al., 2012). Another study also confirmed that LRP1 in the liver can mediate systemic clearance of Aβ. In plasma, the soluble LRP1 is the major transporter of peripheral Aβ. The soluble LRP1 forms a LRP1-Aβ complex by binding of peripheral Aβ, which reduces the concentration of Aβ monomer in plasma, thereby inhibiting plasma free Aβ monomer from re-entering the brain. In AD, LRP1 expression at the BBB is reduced, and the rupture of LRP1 in the circulation results in a decrease in its ability to bind to Aβ. Cell surface LRP1 and circulating LRP1 are targets for drug therapy. Through lifestyle changes, statins can increase LRP1 expression in BBB and liver, and control the risk of AD (Sagare et al., 2012). It has been reported that because the liver has a strong ability to catabolize Aβ and excrete into bile, it has little effect on plasma Aβ levels in general liver function damage (Ghiso et al., 2004; Roher et al., 2009).

#### Amyloid-β monomer clearance in kidney

The kidney participates in the physiological metabolism of Aβ by filtering Aβ from the plasma to the urine. The Aβ excreted by the kidneys is mainly soluble Aβ monomer, because the oligomers and fibers have a large molecular weight and cannot pass through the glomerular filtration. Soluble Aβ is a normal component of human urine, mainly Aβ_40_. The kidney plays an important role in the peripheral clearance of Aβ. A clinical study of chronic kidney disease (CKD) conditions explored the relationship between plasma Aβ levels and cognitive decline, and the results showed that subjects with high plasma Aβ levels had worse cognitive dysfunction scores than those with lower levels (Gronewold et al., 2017). According to the above studies, cognitive function decline in CKD is apparently due to increased plasma and brain Aβ load. Another study found that plasma Aβ levels (mainly Aβ_40_) increased with the progression of CKD and were not affected by hemodialysis, suggesting that the kidney plays an important role in the peripheral clearance of Aβ, and CKD increases the risk of cognitive impairment (Gronewold et al., 2016). At the same time, some studies suggest that hemodialysis can reduce plasma Aβ levels during CKD (Liu et al., 2015). In the case of AD, elevated levels of Aβ in the plasma may be associated with a progression of AD if accompanied by a renal clearance disorder of Aβ caused by impaired renal function. Therefore, improving impaired renal function to reduce plasma Aβ levels can be used as a strategy to prevent cognitive dysfunction.

### Links of amyloid-β in the brain, plasma and peripheral tissues

There is a dynamic balance of Aβ between the brain and plasma, mainly between soluble Aβ. Aβ in the plasma is almost always soluble, mainly monomer, but also some oligomers, but no fiber. Aβ is generally considered to be soluble less than 20-mer and insoluble more than 20-mer. Aβ_40_ oligomers are generally dimers, trimers, and tetramers, and Aβ_42_ oligomers can be dimers, trimers, and tetramers, even consisting of more than 1,000-mer. Aβ in the plasma is mainly Aβ_40_ and Aβ_42_ is also present. Soluble Aβ monomers and oligomers are also present in the brain, and soluble Aβ oligomers form insoluble Aβ oligomers, further forming Aβ fibers, forming plaque deposits, which is one of the pathogenesis of AD.

Soluble Aβ monomers and oligomers can communicate between the brain and plasma through various pathways such as *trans*-BBB transport and CSF. Under physiological conditions, Aβ in the brain and plasma can exchange through the BBB. We have provided a clear evidence that, in a parabiosis model surgically fused an AD transgenic mouse with wild type mouse, the plasma Aβ from a transgenic mouse can penetrate to the wild type mouse and cause AD-like pathology in the brain (Bu et al., 2018). Our studies suggest that plasma Aβ is the important part of Aβ pathogenic process of AD and peripheral clearance of Aβ may be more favorable than the brain for the drug development targeting Aβ. However, the amount of Aβ transported from the brain into the plasma is greater than the amount of plasma transported into the brain. In the case of AD, it has been reported that the amount of Aβ transported to the plasma in the brain is reduced by about 30% (Mawuenyega et al., 2010).

At present, there is still controversy about whether the total Aβ level in the plasma rises or falls in the case of AD. One Study has compared plasma total Aβ, Aβ_40_, and Aβ_42_ levels in AD, mild cognitive impairment (MCI), and normal populations, and no statistically significant differences were found (Toledo et al., 2011). Plasma Aβ biomarkers are closely related to CNS Aβ status, but peripheral tissue has less effect on plasma Aβ. Free plasma Aβ is not a reliable biomarker for measuring the risk and diagnosis of AD. Plasma-soluble Aβ do not reflect the deposition of brain Aβ. This may be because soluble Aβ in plasma is mainly produced by extracellular tissue cells (Lovheim et al., 2017).

Aβ_42_ is produced by the brain into the plasma, so the level of Aβ_42_ in the plasma can reflect the cross-BBB exchange of Aβ. Most studies have concluded that plasma Aβ_42_ levels and plasma Aβ_42_/Aβ_40_ ratios decrease (van Oijen et al., 2006). But another studies suggest that higher Aβ_42_ concentrations can significantly increase the risk of AD (Hansson et al., 2012; Hsu et al., 2017). In addition, other report found that there is no correlation between the concentration of soluble Aβ in plasma and the occurrence of AD (Roher et al., 2009).

Since the detection of soluble Aβ in plasma does not assess the development of AD, current research on plasma Aβ has begun to shift to detect changes in its secondary structure (Nakamura et al., 2018). 15–20 years before the clinical symptoms of AD, the secondary structure of Aβ changed from disordered or α-helix to β-sheet enriched secondary structure. It was found that the change of secondary structure of Aβ peptide is a reliable plasma in severe AD stage (Nabers et al., 2018).

Some experimental studies have found that peripheral clearance can reduce the concentration of Aβ in the plasma, and then guide the outflow of Aβ in the brain through the dynamic balance, thereby reducing the deposition of Aβ in the brain and improving cognitive function (Wang et al., 2017). In another study, intravenous NEP was used to increase plasma Aβ clearance to see if it reduced brain Aβ levels. It was found that plasma Aβ levels in normal and AD mice decreased after 5 weeks treatment, but no significant decrease in Aβ levels in the brain was found (Walker et al., 2013).

The above results demonstrate that the total amount of soluble Aβ in the plasma is fluctuating and has no clear correlation with AD. We speculate that this is mainly because the plasma is dominated by Aβ_40_, which is mainly derived from the production of platelets and peripheral tissues, while the decrease in the amount of Aβ_40_ transferred from the brain is not enough to have A significant impact on the plasma Aβ_40_. In addition, Aβ_42_ in the plasma is mainly derived from the brain, and in the case of AD, the amount of Aβ_42_ entering the plasma from the brain is reduced, and the level of Aβ_42_ in the plasma is lowered. We speculate that there is no significant effect on the total amount of Aβ in the plasma. On the other hand, at different stages of AD, the change of plasma Aβ level is also fluctuating, so the relationship between Aβ level in the plasma and AD may lead to different conclusions. We believe that this needs to be further studied in different stages of AD.

## Structure forms and toxicity of amyloid-β and other diseases besides Alzheimer’s disease

Amyloid-β has attracted much attention for its association with over 30 illnesses (Harrison et al., 2007). Besides AD, Aβ plays a crucial role in other brain disease like CAA and prion disorders, as well as systemic diseases such as osteoporosis and type 2 diabetes mellitus (T2DM). Aβ can deposit in cerebral and peripheral tissues, and aggravate the pathological damage of these diseases, which are all thought to involve structure form changes of Aβ proteins, such as α-helix to β-sheet folding transition.

### Amyloid-β and cerebral amyloid angiopathy

Cerebral amyloid angiopathy is cerebrovascular amyloid deposition, which the pathological changes of CAA are mainly the deposition of amyloid fibers on the middle and outer membrane of the small arteries and capillaries in the occipital and temporal cortex (Wang et al., 2000; Vinters, 2015). The classification of CAA is based on the type of amyloid proteins deposited in the cerebral blood vessels, and Aβ-CAA refers to one type of CAA that mainly deposits amyloid proteins as Aβ. Aβ-CAA is commonly found in elderly individuals and AD patients. Cerebrovascular Aβ originates mainly from the brain and is transported to the vascular wall through a perivascular drainage pathway, where it polymerizes into fibrils on vascular basement membrane through interactions with extracellular components. Aβ in the cerebrovascular is mainly Aβ_40_, and the severity of CAA correlate with the levels of soluble Aβ_40_ in the brain. In the process of vascular Aβ deposition, Aβ_42_ is initially deposited, and later Aβ_40_ is massively accumulated (Yamada and Naiki, 2012). In the past, the incidence of CAA was thought to be related to overproduction of Aβ_40_, a decrease of Aβ degradation, or a decrease in Aβ clearance due to impaired perivascular drainage. Recent study has found that Aβ of CSF increases with normal aging, but both Aβ_40_ and Aβ_42_ of CSF decrease at CAA, which can be used as a biological marker of CAA. Reducing the production of Aβ by inhibiting β-secretase at the early stage can prevent progression of CAA (Schelle et al., 2019). Study has observed that Aβ-CAA occurs after intraperitoneal injection of Aβ oligomers in mice and in some patients with traumatic brain injury. It is speculated that this may be related to Aβ oligomers, which may be attached to metal surfaces and to resist conventional hospital sterilization. This suggests that the pathogenesis of Aβ-CAA may be caused by Aβ oligomers (Jaunmuktane et al., 2015). Further research found that mice with high expression of Aβ_40_ did not find significant CAA pathology, whereas mice expressing Aβ_42_ accumulate insoluble Aβ_42_ and develop CAA. This suggests that the pathogenic oligomer of CAA is likely to be Aβ_42_ oligomer, not Aβ_40_ (McGowan et al., 2005; Watts et al., 2014). Amyloid peptides and proteins in review. Amyloid peptides and proteins in review. FXIIIa colocalizes with Aβ in CAA which the ability of Aβ_42_ to bind FXIIIa is greater than that of Aβ_40_, and that FXIIIa forms unique protein complexes with Aβ that might play an important role in Aβ deposition and persistence in the vessel wall (de Jager et al., 2016).

### Amyloid-β and type 2 diabetes mellitus

Both T2DM and AD are associated with Aβ deposition and insulin signal abnormalities during the pathogenesis. Insulin resistance is found in AD brains, especially in the cerebral cortex and hippocampus. This shows that AD is closely associated with T2DM. On the one hand, islet amyloid polypeptide (IAPP) is a hallmark feature of T2DM patients and involves pancreatic β-cell death (Montane et al., 2012). Studies have shown that IAPP deposition in the brain of AD patients is independent of Aβ (Jackson et al., 2013; Fawver et al., 2014). Inoculation of IAPP aggregation into AD mouse brain can aggravate AD pathology and memory impairment. Mice overexpressing human IAPP showed reduced exploratory behavior and impaired recognition memory, and a wide range of IAPP sediments and inflammatory markers were observed in their brain (Srodulski et al., 2014). The lack of IAPP mRNA in human brain indicates that the IAPP oligomer produced from pancreas enters the brain through blood circulation and promotes the accumulation (Jackson et al., 2013). IAPP oligomers can disrupt the membrane permeability of neurons, induce ROS, and alter Ca^2+^ homeostasis and cell viability in astrocytes and neurons (Janson et al., 1999). Of particular interest is that IAPP has been found to have a typical β-sheet structure that can be aggregated alone or in combination with Aβ to form oligomers or fibers. It has been demonstrated *in vitro* studies that IAPP accelerates Aβ aggregation and the resulting fibers are composed of two peptides (Moreno-Gonzalez et al., 2017).

On the other hand, elevated plasma Aβ levels accompanied by insulin resistance and subsequent deposition of Aβ in the brain were found in AD mice, and the introduction of anti-Aβ antibodies improved insulin sensitivity and glucose regulation (Zhang et al., 2012, 2013; Wijesekara et al., 2017). It was found in AD-T2DM transgenic mice that Aβ can only be deposited in islets in the presence of IAPP. However, no Aβ deposits were found in the islets of AD mice, and no Aβ deposits were found in the islets of T2DM mice, indicating that the Aβ deposits in the pancreas were not generated by pancreatic APP, but possibly from Aβ in the blood circulation, and the accumulation of Aβ in the pancreas was the result of cross-inoculation with IAPP (Wijesekara et al., 2017). Aβ can competitively bind to insulin receptors and stimulate insulin resistance, and Aβ has been shown to inhibit autophosphorylation of the insulin receptor and reduce insulin receptor levels and activity (Ling et al., 2002; Xie et al., 2002; Zhao et al., 2008; De Felice et al., 2009; Bomfim et al., 2012). In addition, Aβ can induce the accumulation of reactive oxygen species, leading to damage to insulin signaling in peripheral tissues (Cai et al., 2015; Kim et al., 2017).

### Amyloid-β and osteoporosis

Epidemiological studies have shown that bone density decreases and the incidence of fractures increases in AD patients, suggesting a link between the two diseases, but this phenomenon is rarely studied (Zhou et al., 2011). APP and Aβ regulate osteoclast (OC) differentiation both *in vitro* and *in vivo*. In AD mice, OC differentiation has an age-dependent biphasic change. The increase in OC differentiation in young mice is mediated by Aβ oligomers and RAGE receptors in bone marrow macrophages (BMMS); the decrease in OC differentiation in aged rats may be due to an increase in soluble RAGE and osteoprotegerin, although the osteoclastic effect is reduced, but this reduces the ability of bone remodeling, thereby increasing the incidence of fracture without significant changes in bone density. This study suggests that APP/Aβ and RAGE act as a common causative agent for AD and osteoporosis (Cui et al., 2011). In addition, another study demonstrated that elevated levels of Aβ_42_ play an important role in the pathogenesis of osteoporosis. The study found that mRNA and protein expression levels of Aβ_42_ and APP were significantly elevated in bone cells of osteoporosis patients and rat models, and showed a negative correlation with bone mineral density. Aβ_42_ is mainly located in the cytoplasmic membrane, cytoplasm and extracellular matrix, which can effectively promote osteoclast differentiation and activation, and abnormal deposition of Aβ occurs in bone tissue. The study concluded that Aβ may be a candidate biomarker for the identification of osteoporosis drug targets (Li et al., 2014).

## Molecular structure-based amyloid-β therapeutic strategy

**FIGURE 7:**
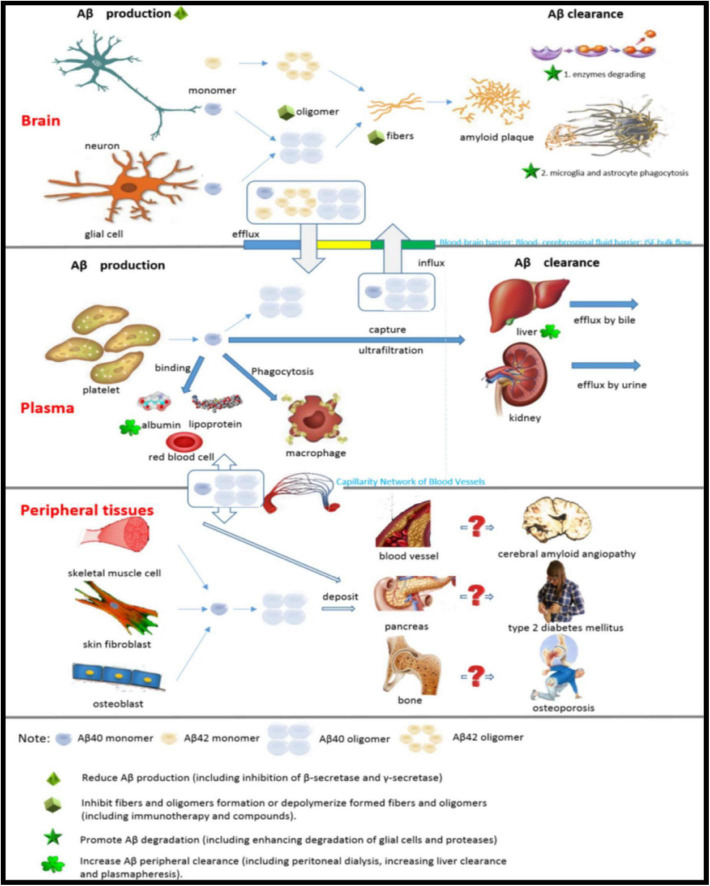
Links of Aβ metabolism in the brain, plasma and peripheral tissues and possible targets for therapy.

The current treatment for AD is aimed at clinical symptoms and does not control the progression of the disease. Links of Aβ metabolism in the brain, plasma and peripheral tissues and possible targets for therapy are shown in Figure 7. Currently, cholinesterase inhibitors and *N*-methyl-D-aspartate receptor antagonists are the only clinically available options. Aβ plaque is the main pathological feature of AD and is the basis of the amyloid cascade hypothesis. The accumulation of Aβ in the brain is an important early factor in the pathogenesis of AD, which ultimately leads to neurodegeneration and dementia. At the present stage, designing drugs and treatments based on the type of Aβ (Aβ_40_ or Aβ_42_) and structural form (fiber, oligomer or monomer) by reducing the generation of Aβ, increasing the clearance of Aβ, or reducing the toxicity of Aβ is the most important direction for the study of prevention and treatment strategies for AD (Table 1).

**TABLE 1:** Therapeutic strategy based on Aβ molecular structure.

Name	Experimental research or clinical trial	Therapeutic strategy for the protein structure of AD	References
1. BACE1 inhibitors		BACE1 inhibitors: inhibiting the process of APP to generate Aβ	
	**Clinical trial** That subjects experienced worsen cognitive function and adverse events (anxiety, sleep, dreams and depression).		Henley et al., 2019
	**Clinical trial** Elenbecestat and umibecestat are still in clinical trials, and have not been found to cause serious cognitive problems.		Selkoe, 2019
2. γ-secretase inhibitor (GSI)		GSI: Decreasing Aβ generation	
	**Experimental research** GSI can not only reduce the generation of Aβ, but also significantly reduce the expression of APP.		Law et al., 2018
	**Experimental research** GSI inhibited β-sheet aggregation and increasing its ability to decompose, reducing caspase-3 and -9 activity.		Carmona et al., 2020
	**Clinical trial** The phase III clinical trial of Semagacestat found that some effects.		Tagami et al., 2017
3. Treatment of oligomer		Promoting oligomer conversion or depolymerization	
	**Experimental research** Phenolic compounds have been found that it can inhibit the oligomerization of Aβ_40_ and Aβ_42_.		Ladiwala et al., 2011
	**Experimental research** Aβ_42_ oligomer antagonists might promote oligomer conversion or depolymerization.		Sinha et al., 2011
4. Degrading Aβ		Promoting the proteases and glial cells to degrade Aβ	
	**Experimental research** Serotonin precursors and their derivatives reduced the Aβ level in the brain of APP/PS1 mice by regulating NEP and improved the memory of mice.		Klein et al., 2018
	**Experimental research** Use of RGFP-966 (a brain-penetrant and selective HDAC3 inhibitor) increased plasma levels of NEP, decreased Aβ at brain and peripheral levels, and improved spatial learning and memory.		Janczura et al., 2018
5. Immunotherapy for Aβ		Immunotherapy for Aβ structural forms, including Aβ monomers, oligomers, and fibers	
	**Experimental research** Studies in AD mice have shown that solanezumab primarily recognizes soluble Aβ monomers and binds to Aβ plaques when used in large doses.		Bouter et al., 2015
	**Clinical trial** In a phase II clinical study of AD patients, solanezumab increased the total Aβ40 and Aβ42 levels in the plasma and CSF of patients, but did not improve the score of the ADAS-Cog14 cognitive scale in phase III trials.		Honig et al., 2018 Doody et al., 2014
6. Aβ monomer clearance in plasma		Aβ clearance in peritoneal dialysis	
	**Experimental research** Intramuscular delivery of p75NTR ectodomain in the brain of AD mices reversed the behavioral deficits and AD pathologies.		Yao et al., 2015 Wang et al., 2016
	**Experimental research** Experiments in AD mice have found that multifunctional magnetite/ceria nanoparticle assemblies reduces Aβ levels in the plasma and brain, and also prevents the spatial working memory deficits.		Kim et al., 2019

BACE1, β-site amyloid precursor protein cleaving enzyme-1; GSI, γ-secretase inhibitor.

### Inhibiting β-site amyloid precursor protein cleaving enzyme-1 to reduce Amyloid-β generation

β-site amyloid precursor protein cleaving enzyme-1 (BACE1) is an essential cleavage enzyme in the process of APP to generate Aβ, and inhibition of BACE1 directly affects AD pathological process. Several years ago, a number of different structurally selective BACE1 inhibitors have been designed and entered the clinical evaluation trial phase (De Strooper et al., 2010; Sambamurti et al., 2011; Yan and Vassar, 2014). Two BACE inhibitors have recently been reported to worsen cognitive function during clinical trials. In atabecestat trial, a BACE inhibitor, Henley et al. (2019) found that subjects experienced worse cognitive function and frequent adverse events such as anxiety, sleep, dreams and depression. Egan et al. (2018, 2019) found that verubecestat worsened cognitive function in another drug trial. Although verubecestat found a decrease in Aβ of CSF and brain in previous tests, it did not find an effect on cognitive function, and found that verubecestat increased the adverse events incidence including anxiety, sleep disorders and depression. For safety, long-term inhibition of this Aβ-producing protease requires the design of more selective drug candidates. Two another BACE inhibitors (elenbecestat and umibecestat) that are still in clinical trials have not been found to cause serious cognitive problems (Selkoe, 2019).

### Inhibiting γ-secretase to reduce amyloid-β generation

The γ-secretase inhibitor (GSI) is a drug that reduces the generation of Aβ by inhibiting γ cleavage in the App membrane. The generation of Aβ requires the cleavage of γ-secretase, so γ-secretase is involved in the pathogenesis of AD and is one of the targets for targeting Aβ treatment (De Strooper and Chavez Gutierrez, 2015). Although some clinical trials have stopped due to lack of clinical efficacy or side effects, animal studies for it are still in progress.

Studies have shown that GSI can not only reduce the generation of Aβ, but also significantly reduce the expression of APP (Law et al., 2018). Another study explored the role of flavonols (mulberry and isoquercetin) and flavanones (hesperidin and neohesperidin) as GSIs, inhibiting β-sheet aggregation and increasing its ability to decompose, reducing caspase-3 and -9 activity (Carmona et al., 2020). Presenilin is a catalytic component of γ-secretase. Studies have investigated whether γ-secretase specificity to substrates in mouse cells expressing human PS1 or PS2 in coordination with PS1 or PS2. As a result, it was found that mouse PS1 plays a major role in γ-secretase cleavage of APP and Notch as compared with mouse PS2. GSI blocks PS2-γ secretase in mice better than PS1-γ secretase, while GSI preferentially targets human PS1- but not PS2-dependent γ-secretase activity (Stanga et al., 2018). Therefore, we should pay attention to these differences when designing GSI and reduce side effects. A study of the causes of failure in the phase III clinical trial of Semagacestat found that some Semagacestat effects were significantly different from those caused by loss of presenilin function. Therefore, Semagacestat may be a pseudo-GSI, and its clinical trials did not really validate the effectiveness of γ-secretase as a strategy for the treatment of Aβ (Tagami et al., 2017). Surprisingly, recent *in vitro* studies have found that low doses of GSI may increase Aβ generation. Low doses of DAPT (a GSI) increased the generation of several Aβ peptides, particularly Aβ_*x–*42_, and increased Aβ oligomers were found in cortical neurons treated with low doses of DAPT (Agholme et al., 2017). Other studies have found rebound effects caused by GSI, such as increased levels of PS1 and other γ-secretase subunits. After the treatment of primary neurons with DAPT or avagacestat (a kind of GSI), the expression of PS1 was significantly increased. After 21 days of taking avagacestat, the expression of PS1 in the brain increased. Long-term taking of avagacestat increased the anxiety-like behaviors in rats (Ono et al., 2012).

### Promoting oligomer conversion or depolymerization

A large number of studies have shown that oligomers are the most toxic species, but it is not clear whether oligomers can be treated as a selective therapeutic target and be remodeled into non-toxic or low-toxic structures with drugs. At present, there are many researches on drug-targeting oligomers and some progress has been made. Studies on phenolic compounds have found that it can inhibit the oligomerization of Aβ_40_ and Aβ_42_, and even the aggregation of Aβ fibers by binding specific amino acid residues on Aβ monomers (Ladiwala et al., 2011). Another study evaluated the conformation specificity and remodeling pathways of small aromatic molecules to Aβ_42_ oligomers and classified them into class I, II, and III molecules according to the different pathways of Aβ_42_ oligomer antagonists. Class I molecules remodel Aβ oligomers into non-toxic large non-pathway oligomers; Class II molecules convert Aβ oligomers into fibers, but do not decompose Aβ fibers; Class III molecules degrade Aβ oligomers and fibers into non-toxic low-molecular substances (Sinha et al., 2011).

Amyloid-β is primarily a pathogenic protein characterized by its own misfolding and aggregation. Therefore, inhibition or regulation of abnormal protein self-folding and aggregation is an effective strategy for preventing and treating Aβ. The study found that a lead compound called CLR01 (Lys specific molecular tweezers) can inhibit the aggregation and toxicity of Aβ by binding to Lys residues, which can destroy the hydrophobic and electrostatic effects that play an important role in the nucleation process, oligomerization and fiber extension of Aβ (Knight et al., 2013; Scheidt et al., 2019).

Once the Aβ_42_ fiber is produced, it can continuously catalyze the formation of new Aβ oligomers on the surface of its fibers through a secondary nucleation mechanism. Recently, Cohen demonstrated *in vitro* that Brichos redirects secondary nucleation to a pathway that forms the least toxic oligomer by binding to the surface of Aβ fibers. This suggests that molecular chaperones can reduce the toxic effects of Aβ by selectively inhibiting secondary nucleation of Aβ (Sinha et al., 2011).

A study by synthesizing a novel cobalt (III) Schiff base complex with a methylamine axial ligand found that it can reduce the formation of β-sheets, destabilize preformed β-sheets and suppress aggregation. This indicates that the Cobalt complex is also a candidate drug targeting Aβ aggregation (Iscen et al., 2019).

### Promoting proteases and glial cells to degrade amyloid-β

Enkephalinase (NEP) is an important catalytic enzyme for the degradation of Aβ monomers and non-pathway oligomers in the brain (Kanemitsu et al., 2003). The expression and activity of NEP is significantly reduced in AD mice and patients, so maintaining and increasing the expression and activity of NEP is a potential strategy for the prevention and treatment of Aβ disease (Bourassa et al., 2019). One Study has shown that serotonin precursors and their derivatives reduce the Aβ level in the brain of APP/PS1 mice by regulating NEP and improve the memory of mice (Klein et al., 2018). Another study demonstrated in the AD mouse model that the use of RGFP-966 (a brain-penetrant and selective HDAC3 inhibitor) increased plasma levels of NEP, decreased Aβ at brain and peripheral levels, and improved spatial learning and memory (Janczura et al., 2018). The above research provides us with new treatment ideas.

In addition, a study has found that the ubiquitin-proteasome proteolytic pathway can degrade APP, thereby reducing Aβ generation (Jung et al., 2015). Another study found that the proteasome disorder involves the UCH-L1, whose mRNA levels are reduced in the hippocampus of the AD brain. Aβ downregulates UCH-L1 in AD brain, which in turn impairs BDNF/TRKB-mediated retrograde signaling, impairing synaptic plasticity and neuronal survival. Therefore, inhibition of Aβ downregulation of UCH-L1, protection of synaptic plasticity and neuronal physiological function is one of the therapeutic strategies that can be explored (Poon et al., 2013).

The study found that Sodium ludin (NaR) not only increased microglia clearance of Aβ by increasing the expression of receptors associated with microglia phagocytosis, but also promoted the transition from anaerobic glycolysis to mitochondrial oxidative phosphorylation, providing microglia with sufficient energy to clear Aβ. Thus, NaR can reduce neuroinflammation, enhance Aβ clearance, reduce synaptic plasticity damage, and reverse spatial learning and memory impairment. This suggests that NaR is a potential therapeutic agent for AD (Pan et al., 2019).

### Immunotherapy for different amyloid-β structural forms

Immunotherapy for Aβ has been a research hotspot, although great progress has been made, it is far from meeting the needs of clinical application. Early immunotherapy was simply targeting Aβ without distinguishing between different structural forms, leading to reduced levels of Aβ in the brain but no improvement in cognitive dysfunction in AD mice and patients, or serious side effects. Targets of current immunotherapeutic research have turned to specific toxic Aβ structural forms, including Aβ monomers, oligomers, and fibers. The antibody targeting Aβ monomer currently under investigation is mainly Solanezumab, a humanized, IgG1 monoclonal antibody that targets the Aβ_13–28_ amino acid residue sequence. Studies in AD mice have shown that solanezumab primarily recognizes soluble Aβ monomers and binds to Aβ plaques when used in large doses (Bouter et al., 2015). In a phase II clinical study of AD patients, solanezumab increased the total Aβ_40_ and Aβ_42_ levels in the plasma and CSF of patients, but did not improve the score of the ADAS-Cog14 cognitive scale in phase III trials (Doody et al., 2014; Honig et al., 2018). The toxicity of Aβ oligomers has the strongest evidence supported by a large number of studies, so monoclonal antibodies targeting Aβ oligomers are a research hotspot of immunotherapy. Crenezumab is a humanized anti-Aβ monoclonal IgG4, which has a strong affinity for the pentamers and hexamers of Aβ. Injection of Crenezumab into the brain of mice does not cause significant inflammatory changes in the brain of AD mice. However, no significant improvement in cognitive impairment was observed (Fuller et al., 2015; Zhao et al., 2017). In a small sample of clinical studies, it was found that Crenezumab can improve cognitive impairment in patients with mild AD (Cummings et al., 2018). CAD106 is an active immunization vaccine consisting of multiple copies of Aβ 1–6 amino acid residues coupled to an adjuvant carrier. The antibody induced by CAD106 mainly binds to the oligomers and monomers of Aβ and blocks its toxicity, and has obvious adverse reactions. However, it does not improve the behavioral or cognitive dysfunction in patients with AD, suggesting that CAD106 is not effective in the treatment of AD (Winblad et al., 2012; Vandenberghe et al., 2017).

Still other monoclonal antibodies target both Aβ oligomers and fibrous structures such as Gantenerumab and Aducanumab. Gantenerumab is a monoclonal IgG1 antibody that binds to the amino terminus and central region of Aβ. It is found in AD mice to have a much higher affinity for Aβ oligomers and fibers than monomers and to reduce Aβ plaques in the cerebral cortex (Bohrmann et al., 2012). Aducanumab is a recombinant human IgG1 antibody that recognizes the Aβ 3–7 amino acid residue sequence, mainly binding to Aβ oligomers and fibers. The study found that Aducanumab can reduce the size of Aβ plaque in the brain of young mice, but has no significant effect on Aβ plaques in the brain of aged mice and AD patients, Aducanumab can Reduces Aβ load in the brain and demonstrates an improvement in cognitive and clinical function for the first time in a dose- and time-dependent manner (Sevigny et al., 2017).

### Therapeutic strategy for amyloid-β monomer clearance in plasma

A study has observed whether peritoneal dialysis can reduce the pathological characteristics and cognitive impairment of AD, and the results showed that peritoneal dialysis reduced the Aβ level of plasma and brain inter-tissue fluid and Aβ deposition, and improved the behavioral defects of AD mice (Jin et al., 2017). This suggests that peritoneal dialysis is a method of treatment for AD.

Neurotrophin receptor p75 (p75NTR) mediates Aβ-induced neurodegenerative signals, and its extracellular domain (p75ECD) is a physiological protective factor against Aβ in AD. p75ECD is significantly reduced in brain tissue and CSF in AD patients and mices. Intramuscular delivery of p75NTR ectodomain before or after Aβ deposition in the brain of AD mices reversed the behavioral deficits and AD pathologies, such as Aβ deposit, Tau phosphorylation and suppressing β-secretase expression and activities. Peripheral administration of p75ECD represents a promising strategy for AD treatment (Yao et al., 2015; Wang et al., 2016).

Plasma albumin inhibited the growth of Aβ_40_ and Aβ_42_ fibers, delayed the formation of fibers, and reduced the total amount of fibers (Stanyon and Viles, 2012). In 2009, a small sample of patients with mild-to-moderate AD was followed up for 1 year. The results showed that plasma Aβ levels were correlated with plasmapheresis, and their cognitive scores were stable during the 1-year follow-up (Boada et al., 2009).

However, the efficacy of this approach may be affected by BBB’s effect on the transport of Aβ in the brain and the entry of Aβ produced by other organs into the serum. The influx of ISF into CSF is another major pathway of Aβ clearance. Although the concentration of albumin in CSF is much lower than that in plasma, CSF mixing with ISF is not hindered by the highly selective barrier and Aβ in CSF is not directly exchanged with the peripheral Aβ, so Aβ in the two pools is more directly exchanged. Albumin replacement in CSF can be used as an alternative to clearing Aβ in the brain (Menendez-Gonzalez and Gasparovic, 2019).

Amyloid-β can be captured by erythrocyte immune adhesion and subject to complement-mediated clearance from the peripheral circulation, but these mechanisms are deficient in AD. Aβ dose-dependently activated serum complement. Studies have found that binding of antibodies to Aβ can significantly increase the activation and opsonization of complement, and subsequently enhance the capture of Aβ by red blood cells and macrophages, and promote the peripheral clearance of Aβ. This indicates that it is a viable pathway for peripheral clearance of Aβ (Brubaker et al., 2017; Crane et al., 2018).

A new extracorporeal Aβ cleansing system, multifunctional magnetite/ceria nanoparticle assemblies, can remove Aβ from the plasma its reactive oxygen species. Experiments in transgenic mice have found that it reduces Aβ levels in the plasma and brain, and also prevents the spatial working memory deficits, suggesting that the method can be used for AD prevention and therapy (Kim et al., 2019).

Alzheimer’s disease patients are often accompanied by vascular risk factors and experimental studies have shown that hypertension, diabetes, and high cholesterol can increase Aβ deposition in the brain (Li et al., 2011; Zhu et al., 2014; van der Kant et al., 2019). In several prospective clinical studies in China, 837 patients with MCI have demonstrated that controlling vascular risk factors can prevent AD (Li et al., 2011). In patients with vascular risk factors, controlling vascular risk factors can reduce the risk of AD (Jiao et al., 2015).

## Conclusion

Hardy and Higgins (1992) proposed AD the pathogenic mechanism of protein cascade hypothesis, Aβ has become the most popular molecule in AD research. A large number of studies have been done on the specific pathogenesis of Aβ, which has a profound understanding of the pathogenesis of AD, but it has not yet fully revealed the pathogenesis caused by Aβ. With the development of structural biology technologies such as sNMR and Cryo-EM, the molecular structure of Aβ has been analyzed at the atomic level, which provides a new and more refined perspective for understanding the role of Aβ in the pathogenesis of AD and developing disease-modifying drugs by targeting Aβ.

## Author contributions

HY and JL developed the review’s concept and structure and contributed to writing this review. XL, LM, and MH participated in some of the writing. HZ and RZ revised and finalized the review. All authors read and approved the final manuscript.

## Abbreviations

AD: Alzheimer’s disease
Aβ: amyloid-β
sNMR: solid-state nuclear magnetic resonance
Cryo-EM: cryo-electron microscopy
APP: amyloid precursor protein
C_83_: α secretase C-terminal fragment
sAPP_β_: soluble APP_β_
C_99_: β secretase C-terminal fragment
AICD: APP intracellular domain
BBB: blood–brain barrier
HMGB1: high mobility group protein-1
LTP: long-term potentiation
LRP1: low density lipoprotein receptor-related protein-1
RAGE: receptor for advanced glycation endproducts
ISF: interstitial fluid
CKD: chronic kidney disease
MCI: mild cognitive impairment
OC: osteoclast
T2DM: type 2 diabetes mellitus
IAPP: islet amyloid polypeptide
BACE1: β-site amyloid precursor protein cleaving enzyme 1
GSI: γ-secretase inhibitor
NEP: enkephalinase
UCH-L1: ubiquitin C-terminal hydrolase L1
p75NTR: neurotrophin receptor p75
p75ECD: neurotrophin receptor p75 extracellular domain.
